# Perceptions and experiences of patients attending an opioid substitution clinic in South Africa

**DOI:** 10.4102/sajpsychiatry.v28i0.1936

**Published:** 2022-12-09

**Authors:** Abdul K. Domingo, Sonja Pasche, Lucy Jarvis, Lize Weich

**Affiliations:** 1 Department of Psychiatry, Faculty of Medicine and Health Sciences, Stellenbosch University, Cape Town, South Africa; 2 Department of Psychology, Faculty of Arts and Social Sciences, Stellenbosch University, Stellenbosch, South Africa

**Keywords:** opioid substitution therapy, opioid use disorders, methadone, buprenorphine, patient experiences, South Africa

## Abstract

**Background:**

Opioid substitution therapy (OST) is endorsed as the recommended treatment for opioid use disorders. Opioid substitution therapy is not widely used in South Africa, so little is known about its perceived clinical utility in this setting. There is also a paucity of qualitative research that explores the subjective experiences of patients using OST.

**Aim:**

To explore patients’ perceptions and experiences attending a South African OST outpatient clinic (OST-OC).

**Setting:**

The OST-OC at Stikland Psychiatric Hospital, Cape Town, South Africa.

**Methods:**

We conducted a qualitative study using semi-structured interviews with eight participants who had been attending the OST-OC for at least 6 months. Transcripts were analysed using Atlas.ti software and thematic content analysis was used to identify themes.

**Results:**

Patients stated that OST helped them to regain and maintain a stable lifestyle. Autonomy and agency, the therapeutic relationship and family support were perceived as contributing to successful patient outcomes. The preference for methadone and buprenorphine treatment depended on individual experiences. Patients valued kindness from staff members but reported that improved interactions with some nonclinical staff could better facilitate treatment. Challenges experienced included stigma and cost.

**Conclusions:**

This study offers insights about OST that are pertinent to low- and middle-income countries. Reducing the cost of OST, collaborative decision-making between staff and patients, and a non-judgemental attitude by clinical staff were recognised as important factors for optimised service delivery.

**Contribution:**

Understanding patients’ experiences of OST in a South African setting will allow for future policy development for the treatment of opioid use disorders in similar settings locally and abroad.

## Introduction

Opioid dependence is increasingly contributing to the global burden of disease.^1,2^ South Africa has one of the highest disability-adjusted life years (DALYs) for opioid dependence (312.2 per 100 000).^1^ High levels of mortality and morbidity in the sub-Saharan region are partly attributed to the lack of access to opioid substitution therapy (OST), needle and syringe programmes and medical care for people with opioid dependence.^2^ Despite poor outcomes,^3,4^ many countries continue to promote abstinence-based therapies as a first-line treatment for opioid dependence.

Extensive evidence demonstrates that access to OST reduces death rates and improves quality of life.^5,6,7^ Opioid substitution therapy is endorsed as the recommended treatment for opioid use disorders; however, it is not widely used in South Africa because of the high cost of the treatment,^8^ which means that for the majority of people in South Africa, OST can be accessed only by those who can afford treatment at private clinics. According to the Global State of Harm Reduction Report,^8^ there are 11 OST projects being implemented in four cities across South Africa. Since 2017, the City of Tshwane has partnered with the Gauteng Department of Health and the University of Pretoria to provide OST in central Pretoria and Tshwane townships.^9^ Stikland Hospital is a tertiary level psychiatric hospital located in a suburb of Cape Town, South Africa, and provides both inpatient and outpatient services. An OST-Outpatient Clinic (OC) was initiated in 2011 as a means of offering evidence-based treatment at a lower cost to patients with known heroin use disorders.

Little is known about the perceived clinical utility of this treatment in the South African setting and there is a paucity of qualitative research that explores the subjective experiences of patients using OST. A previous South African study assessed factors influencing retention rates among low-income individuals provided with methadone for a heroin use disorder.^10^ This study concluded that the use of a harm reduction approach and a restorative justice paradigm resulted in higher retention rates. Furthermore, a systematic review assessing patients’ perspectives to accessing OST highlighted negative treatment perceptions, cost, stigma and a lack of availability as the most common barriers to accessing OST.^11^ Access to patient perspectives on OST services is vital to improve the quality of local services and to inform expansion of services. This information may also inform future policy around the implementation of treatment services for people with opioid use disorders in South Africa and other low- and middle-income countries. Therefore, the primary aim of this research was to explore patients’ perceptions and experiences of attending the OST-OC at Stikland Hospital.

## Research methods and design

### Study design

This cross-sectional qualitative study was conducted at the OST-OC at Stikland Psychiatric Hospital (Cape Town, South Africa) between February and May 2017. The researchers used a phenomenological approach^12^ to explore the subjective experiences of patients receiving OST.

### Study setting

Stikland Hospital is one of the three public psychiatric hospitals in the Western Cape province of South Africa. This OST-OC provides services to patients from all areas across the Western Cape. This clinic is available over a 5-h period once a week. Clients are assessed and managed by either a specialist registrar or psychiatrist. Patients are primarily managed for their opioid use disorder, although comorbid psychiatric disorders are concurrently managed.

### Study sample

Patients who had been attending the OST-OC for a period of at least six months were invited to participate in the study by their treating physician. A duration of six months attendance at the clinic was chosen because clients often require several months to stabilise on adequate doses of medication, to manage difficult psychosocial circumstances and to develop a rapport with their doctors. An audit completed at the time of the study found that 12 individuals within the OST clinic were eligible to participate in the study. All 12 individuals were seen face to face by the primary investigator of this study, of whom eight agreed to participate. The other four patients declined to participate because of work commitments.

### Data collection

After providing written informed consent, participants were first asked to complete a questionnaire which captured their socio-demographic information and details regarding their substance use history and level of satisfaction at the OST-OC. Each participant was then interviewed by a psychiatrist not currently involved in their treatment (Appendix 1). The individual, semi-structured interviews were approximately 45 min – 60 min long and were audio-recorded. Recordings were transcribed verbatim by a professional service provider. All transcripts were checked for accuracy.

### Data analysis

Interview transcripts were thematically analysed via an inductive approach,^13^ and themes were identified at a semantic level. The researchers were guided by Braun and Clarke’s phases of thematic analysis.^14^ All transcribed interviews were initially read and re-read to obtain an overall impression of the content and to identify pertinent patterns of experience. Codes were used to document identified patterns and were later combined into overarching themes. A subset of the interviews was inductively and independently coded by two researchers. Their findings were then discussed and further refined. Both researchers then continued to analyse all interviews for any additional significant themes. Atlas.ti software version 7 was used to assist with coding and the identification of themes. As a means of further validating the accuracy of these findings, the identified themes were discussed and approved by the psychiatrist who interviewed the participants, as well as four of the participants. All five individuals agreed with the established themes and no amendments were suggested.

## Results

The demographic characteristics of the eight participants who agreed to take part in this study are summarised in Table 1. The mean (standard deviation [s.d.]) age of the participants was 32.4 (s.d. 2.2) years and the mean age of education was 11.4 (s.d. 1.4) years. The mean age of first drug use was 14.4 (s.d. 2.2) years. The mean OST-OC satisfaction score was 9 (s.d. 0.82) out of a maximum score of 10, indicating very high satisfaction.

**TABLE 1:** Demographic characteristics of participants (*n* = 8).

Variable	Mean	s.d.	*n*	%
Age (years)	32.4	2.2	-	-
Age at first drug use (years)	14.4	2.2	-	-
**Gender**				
Male	-	-	4	50.0
Female	-	-	4	50.0
**First drug used**				
Cannabis	-	-	4	50.0
Heroin	-	-	2	25.0
Codeine	-	-	1	12.5
Alcohol	-	-	1	12.5
**History of ever injecting**				
No	-	-	3	37.5
Yes	-	-	5	62.5
**Occupation**				
Employed	-	-	3	37.5
Studying	-	-	2	25.0
Unemployed	-	-	3	37.5
Number of years of education	11.4	1.4	-	-
OST-OC satisfaction score	9.0	0.82	-	-

s.d., standard deviation; OST-OC, opioid substitution therapy-outpatient clinic.

Based on the thematic analysis of interview transcripts, seven key themes emerged.

### Theme 1: Institutional considerations

Participants described the service they encountered at Stikland Hospital as being professional and organised. The facility was said to be clean and neat, which participants felt reflected the level of care this facility provided to their patients. Patients reported feeling welcomed, and several patients particularly appreciated being greeted by clinical and nonclinical staff members when walking by. Patients interpreted this gesture as showing care and kindness:

‘The neatness and everything, and uhm, the staff they were friendly and they were like professional, and I actually didn’t wait all that long.’ (Participant 3, female, DOR: 07 March 2017)

‘… you can see they have been doing it up nicely and, and as a patient you perceive it as if they’re going through the trouble to make it pleasant for you, so it shows care.’ (Participant 1, male, DOR: 14 February 2017)

Most of the participants felt the amount of money being charged was reasonable for the services obtained, while some participants mentioned certain tensions regarding payment. This seemed to relate to difficult interactions with an administration clerk. Participants expressed some distress about this employee’s constant reminders regarding outstanding fees and the manner in which this information was delivered. Participants stated that these interactions made them question returning for treatment. They also reported a sense of relief when they learned this employee had been moved away from the clinic:

‘I wouldn’t say they were harassing me for the money, but it was, they were constantly reminding me, so I was thinking like ok, well I almost felt like I couldn’t come this week if I don’t pay it, because they [*are*] going to give me these bills and these slips and stuff like that.’ (Participant 7, male, DOR: 09 May 2017)

### Theme 2: Family support

While participants appreciated the support from clinical staff, support from their family emerged as the most important support structure during their recovery. One participant shared that time with his doctor was ‘an hour out of thousands of hours’, and that they needed outside support for their treatment to be successful. Participants indicated that their family members assisted with referral to the clinic, brought them to their appointments and provided ongoing emotional support:

‘So they have been supporting me through this long road, uhm, but if it wasn’t for them and their prayers, uhm, I wouldn’t have been here today.’ (Participant 4, female, DOR: 14 March 2017)

All participants relied on family members to initially fund their OST and many reported being encouraged by their family to remain on it:

‘Ja, and I think, and they’re willing to pay, they say they’re uhm willing to pay for it. I know it is expensive, I think they would rather have me on the Methadone than anywhere else.’ (Participant 2, female, DOR: 07 March 2017)

In addition to providing emotional and financial support during recovery, families were also described as being a strong motivator for seeking and staying on treatment. While most participants described their decision to abstain as their own, many described wanting sobriety to maintain their relationships with family members and avoid disappointing them:

‘I just thought, ok, here’s my chance, let me just use it, I felt like I didn’t want to disappoint him, I felt I owed it to my mom and my grandparents, and whoever else, so I took the opportunity and here we are.’ (Participant 7, male, DOR: 09 May 2017)

### Theme 3: Agency and independence

Despite the external influence of family members, participants described their recovery as being successful because it was something that they themselves were ready for and wanted. Their families provided them with the option of seeking treatment, but it was their own personal decision to make. This represents an assertion of independence and personal agency.

However, the act of entering treatment and requesting help was described as a difficult process and served as an acknowledgement of their reliance on treatment, which made them feel vulnerable. Participants reported on the difficulties associated with giving up a substance that had helped them regulate their emotions and thoughts. Facing life without their substance and allowing another individual to take over their care was said to be very difficult. Therefore, the initiation of treatment was also associated with a loss of agency and independence:

‘As an addict the person that’s trying to help you is actually the enemy because he wants to take you away from what you enjoy and love. So, there’s like this battle of wills with, or for me it was always just like “I’m, I’m stronger than this, you guys won’t fix me”, “I will do what I want to do” and “I will stop when I want to stop.” That, that kind of battle of wills, or you don’t, you know you don’t want to, … you know like allowing somebody to help you it’s like you know telling yourself you’re helpless. It’s not, no person likes to think of themselves in the negative, even if you know you have a problem you still a normal human being.’ (Participant 1, male, DOR: 14 February 2017)

The sense of agency and independence seemed to be restored during the treatment process. All participants expressed shared decision-making regarding treatment dosage and duration. Clients felt that they had the final say regarding changes in medication doses, but appreciated being guided by their doctors, whom they felt they could trust.

‘Well like for instance, the doctor will ask me like, “are you comfortable with your dose?” Like it’s not “you have to get off it, now!”’ (Participant 3, female, DOR: 07 March 2017)

### Theme 4: Therapeutic relationship

The participants valued the relationships they formed with the clinical staff members. Participants appreciated their physicians’ nonjudgmental attitudes and ability to convey genuine interest and care during consultations. Participants described a sense of being listened to, and how important this was for them:

‘This is the one place I feel I can be totally honest … I’m not going to be judged, and then we will actually sit and talk about what happened and think about how we can, how I can, what I can do and what they can help me with not to use again.’ (Participant 2, female, DOR: 07 March 2017)

The good therapeutic relationship and rapport that formed between clinician and patient facilitated easier discussions regarding ongoing substance use, lapses and relapses, and motivated clients to want to do better. Not being blamed for past lapses allowed patients to view their substance use in an objective manner, promoted honest discussions and allowed patients to follow-up without fear of judgement:

‘… also they will make you feel comfortable with “it’s fine to relapse, but you have to work on it ”. They will not make you feel “okay, you relapsed, you’re a bad person, please just go, you can’t do this, this is not for you, recovery is not for you”. They encourage you until you get to that point where you want to do it yourself.’ (Participant 4, female, DOR: 14 March 2017)

Participants described their time with their doctor as being meaningful and important, and explained that it contributed to a better understanding of their addiction and matters related to maintaining their recovery. Having this time and space was therapeutic and patients reported looking forward to their appointments:

‘So this is my, my recovery time, is speaking …. I started to, you know, uhm clarify a lot of things that I feel and make sense of it. And, and always afterwards I felt like I, certain things would click in place and my understanding was better. So uhm, that is the routine it gave me.’ (Participant 1, male, DOR: 14 February 2017)

### Theme 5: Opioid substitution therapy offers stability

Participants agreed that OST provided them with a sense of stability, not only in terms of cravings and lapses, but also in terms of being able to progress in other aspects of their lives:

‘…now I can actually, with the medication, and exercise, and things like that I can actually enjoy regular stuff.’ (Participant 3, female, DOR: 07 March 2017)

Many participants described several failed recovery attempts before choosing OST, and some expressed a sense of deep regret at not making use of it earlier:

‘Ja [*yes*], I think I hadn’t been on methadone before so I think that also was a bit different to other places, where they just think methadone is bad, you can’t stay on it. And I’ve also read a lot about how it actually works. In certain cases where, like me, I could never stay clean, never stop using, but the methadone it helps so much. It’s still helping, it’s just, part of me was like I wish I’ve tried something like this years before.’ (Participant 2, female, DOR: 07 March 2017)

Opioid substitution therapy facilitated recovery by decreasing cravings and ‘taking the edge off’. Dosing was also limited to once daily as opposed to several times a day with heroin. Despite the costs associated with the medication, participants felt they were able to save money by using OST. One participant reported that his decision to use OST was because it was the most ‘practical and economical decision’ for him as it allowed him to continue with his employment and complete his studies, as opposed to taking time off to be admitted to an inpatient rehabilitation centre:

‘So, I don’t earn like a massive salary, but it’s uhm, but uhm I think the positives outweighs the negatives. I would rather be spending a thousand rand a month on suboxone and I’m clean, then spending everything I have every day on heroin.’ (Participant 1, male, DOR: 14 February 2017)

### Theme 6: No distinct preference for type of opioid substitution therapy medication

Four participants reported using methadone and four used suboxone as their current treatment. Each participant described their choice of medication as being the correct treatment option for themselves.

Two participants described being initiated on OST while pregnant and had been advised by their doctors to use methadone. Some participants described methadone as their preferred treatment because it had eliminated cravings which promoted abstinence. One participant described a history of concurrently using methadone, heroin and methamphetamines, but had maintained abstinence on methadone since attending the OST clinic:

‘… it’s just that craving is so much less on the methadone. Where I found suboxone didn’t help the cravings for me, the couple of times that I took it. But the methadone really, almost feels like, I don’t know, I won’t say it calms me down, but my thoughts have been, just much better on it.’ (Participant 2, female, DOR: 07 March 2017)

Other participants preferred suboxone as it was not possible to obtain euphoria from this medication, or any illicit opioid while on treatment, which discouraged any further use of heroin. Those on suboxone feared withdrawal from methadone, which they heard was quite severe:

‘I use my suboxone for 30 minutes, in my mouth and then I don’t have any problems, and you can’t use on top of it, that’s the main reason I stick to suboxone.’ (Participant 5, male, DOR: 14 March 2017)

### Theme 7: Challenges experienced

Many of the participants highlighted stigma as a challenge during their recovery. Participants expressed that Stikland Hospital had a certain stigma associated with it because they believed that only people with a severe mental illness attend services there:

‘Well, uhm, my parents always threatened me saying, “you going to go to Stikland” [*laughs*] but I was like “I’m not crazy.”’ (Participant 3, female, DOR: 07 March 2017)

Participants described their experiences of stigma related to addiction. Medical practitioners seen outside of this clinic were described as having little empathy towards those with addiction and did not provide the same level of care.

Stigma was found to also be associated with their choice of treatment. Healthcare professionals, including those linked to addiction treatment services, were reported to have warned against the use of OST. These professionals believed that OST is dangerous and that it should be used for only a short period of time to avoid substituting one addiction for another. Participants receiving OST were made to feel that sobriety can only be achieved without the use of these medications:

‘… my doctor outside, he said like, “you know it’s a short-term thing,” and um, he’s got his different views about it, and he’s entitled to that, that’s ok, but, um, I said to him, “listen it’s got me this far, and I am not going to give it up now …”’ (Participant 7, male, DOR: 09 May 2017)

The costs incurred for travelling, hospital payments and funding OST were described as a major challenge by all participants. Many described the amount required by the hospital as being an affordable amount, especially when compared to other institutions. However, many of the participants often lacked money to pay for treatment:

‘I was bringing myself, by public transport, so they were like sort of ok, you need to pay this, you need to pay that, and the money was starting to slowly build up […] and at that time I didn’t have the money to pay for it.’ (Participant 7, male, DOR: 09 May 2017)

As highlighted previously, all participants relied on assistance from family to fund the costs of their medication. Participants agreed that OST was a cheaper option compared to ongoing heroin use, but some stated that funds were not always available to guarantee a month’s worth of treatment. Some described choosing a lower dose of treatment to make ongoing treatment a possibility:

‘Like really expensive, and I would have like to use 8 mg, because the amount that I use, and my history, I can’t afford it, so, I asked for 6[*mg*].’ (Participant 5, male, DOR: 14 March 2017)

Participants expressed their frustration and fear around missing appointments and the difficulty in obtaining a prescription for their medication. Opioid substitution therapy is considered a schedule six medication and therefore requires an original script. Most of the participants expressed their fears relating to the availability of doctors outside of clinic times and the costs of seeing a private medical practitioner to obtain a script:

‘I just think that maybe if there’s a doctor, not on duty, let’s say for instance docter … not there and you maybe missed your appointment with that story I spoke to you earlier on about. Maybe if they have someone on call, that could maybe just help you straight away.’ (Participant 7, male, DOR: 09 May 2017)

## Discussion

This study explored the experiences of eight individuals attending an OST-OC within a psychiatric hospital in South Africa. The results showed that experiences and perceptions were overall positive.

Descriptions of positive experiences included time spent with staff members and perceptions regarding the clinic. The cleanliness of this clinic was viewed as the institution demonstrating interest in the care of its clients. Cleanliness has been described as encompassing the appearance of the environment, physical cleanliness and staff behaviour, and is considered to be a key influencing factor for patients when choosing a hospital.^15^ The way a former employee discussed outstanding fees and costs was flagged as being poorly managed at this clinic and caused significant distress among patients.

The support provided by family members was identified as one of the main factors that made recovery possible for these participants. They described their families as the main motivators for recovery, with family support regarded as more important than that provided by the staff at the OST-OC. Families were heavily relied upon to finance this currently expensive form of treatment. Higher functional social support at intake has been found to be a positive predictor of retention in treatment,^16^ and research has consistently confirmed the efficacy of family involvement in drug addiction treatment.^17^

Agency was described as a complex and particularly important theme. Firstly, participants described their agency in terms of wanting treatment and sobriety, that this decision was not one that had been forced upon them. A loss of agency, however, occurred when taking on the role of being a patient, resulting in a strong sense of vulnerability and dependency on others. Patient motivation is already known to be an important factor in addiction treatment,^18^ and internal motivation has been found to be a predictor of lower relapse rates within a methadone treatment setting.^19^ There is, however, considerably less emphasis and acknowledgement placed on the vulnerability and loss of agency experienced by individuals taking on the patient role.

Loss of agency may be buffered by the therapeutic relationship formed between clients and treatment staff, as well as the sense of autonomy and shared decision-making. The therapeutic relationship served as a means of clarifying current concerns and validating the progress made. Empathy shown by doctors was especially important in the wake of addiction-associated stigma which participants revealed to be prevalent in circles outside of psychiatric care.

An early therapeutic alliance has been shown to be a consistent predictor of engagement and retention in drug treatment.^20^ Client-centred therapy in methadone clinics has been found to produce positive therapeutic alliances and promote ongoing treatment.^21^ These findings seem to indicate that the mere act of dispensing OST to an opioid-addicted individual should not be considered as the only factor required for positive treatment outcomes.

Despite half of this cohort using a partial opioid agonist (suboxone) and the other half using a full opioid agonist (methadone), all participants viewed their choice of treatment as being the preferred option for themselves. Some patients preferred suboxone because it prevented the experience of euphoria from other opioids, while some preferred methadone as they felt it provided them with a sense of relief from cravings not previously experienced on other treatment. These findings are in line with other qualitative studies investigating the use of these medications.^22,23^ All participants described OST as assisting them in their recovery through decreased cravings for heroin and the practicality of once daily dosing of these medications. This newfound sense of stability provided an advantage over past attempts at recovery and allowed them to progress in other areas of their lives. Some treatment providers outside of this clinic were, however, described as actively discouraging the use of these medications, citing fears of it causing harm or an ongoing addiction to this form of treatment.

### Limitations

As with all qualitative research, causality cannot be determined in these findings. The researchers involved in the care of these participants may have biased the participants’ views. The researchers’ own subjectivity when analysing the transcripts may have also influenced the themes identified. As a means of limiting this bias, researchers who were not involved with the care of these clients were asked to act as the interviewer and to act as a second coder. This sample represents individuals who have managed to remain in treatment for a period of at least six months and therefore may not have provided a full range of patient perspectives. Future studies should consider a longitudinal assessment and include individuals who recently started OST and individuals who have dropped out of treatment.

## Conclusion

While retention rates within low-income clinics providing methadone have previously been assessed,^10^ this qualitative study has for the first time documented the experiences of those attending an OST clinic in South Africa, a country where OST maintenance is not government subsidised. The previous study by Marks et al.^10^ was also conducted at a low threshold clinic. The participants described OST as providing a clear advantage during recovery, but that one type of medication cannot be assumed to be ideal for all. Perceived quality of care was dependent on the cleanliness and appearance of the institution and the non-judgemental and supportive interaction with both clinical and nonclinical staff members. Participants reported that family was an important motivator for their recovery, and provided the financial assistance to afford OST. Participants said they experienced an initial loss of agency and autonomy when initiating treatment, but over time regained a sense of control as OST contributed to a sense of stability in their daily lives. Despite the evidence supporting its efficacy, OST. Participants to be an expensive form of treatment in South Africa and is limited to those with the financial means and social support to fund it. The experiences described by these participants are particularly valuable as they offer insights pertinent to low- and middle-income countries and may guide future treatment centres wishing to offer this evidence-based form of treatment. This study has also highlighted the need to further investigate what other services should be incorporated with the provision of OST.

## Appendix 1

Outline for semi-structured interview:

1. How have you come to be part of the clinic? What were you hoping to achieve?
  Points to be discussed:
  - How easy was it to gain access to this clinic?
  - Why have you chosen to utilize OST in the management of your addiction?
2. What are your thoughts regarding the medication you are using?
  Points to be discussed:
  - Beliefs and opinions regarding both methadone and suboxone.
  - What guided their decision regarding current treatment choice.
  - Benefits and negatives while treated on OST (NB: thoughts on costs).
  - Beliefs around duration of use.
  - Beliefs around dosing.
3. How have you experienced the Stikland OST Clinic?
  Points to be covered:
  - How has your life changed on OST.
  - Positive and negative experiences at this clinic.
  - Relationship with treatment providers (doctors, nurses, admin staff).
  - Has the patient felt involved in decision making/perceived participation.
  - Do they wish to be involved in decision making or wish to have a more passive involvement.
  - Experience of counseling at the clinic.
  - Experience of OST from another service/clinician and how does Stikland OST compare to this.
4. How would you rate the quality of this service?
  Points to be covered:
  - Elements of treatment provided that is of benefit/has been helpful. (What about the clinic does the client believe makes it successful).
5. What would make the clinic better?
  Points to be covered:
  - Identify any unmet needs (additional services).
  - Problems associated with the running of the clinic – eg: waiting times, fees, rotating doctors, frequency.
6. Have you experienced any stigma/discrimination regarding your decision to utilize Stikland OST clinic?
  Points to be covered:
  - Stigma regarding decision to use OST from within Stikland Hospital, substance treatment services, pharmacies, family, other.

(Explore patient’s own feelings around being in recovery and utilizing OST)
